# Time Does Matter: Reduced Internal Medicine Clerkship Clinical Experiences Due to COVID-19

**DOI:** 10.7759/cureus.32445

**Published:** 2022-12-12

**Authors:** Gloria M Coronel-Couto, Amalia M Landa-Galindez, Melissa L Armas, Rodolfo Bonnin

**Affiliations:** 1 Department of Translational Medicine, Herbert Wertheim College of Medicine, Miami, USA; 2 Department of Medical Education, Nova Southeastern University Dr. Kiran C. Patel College of Allopathic Medicine, Fort Lauderdale, USA; 3 Department of Psychiatry, Herbert Wertheim College of Medicine, Miami, USA

**Keywords:** covid-19 retro, nbme shelf, clerkship length, covid-19, internal medicine clerkship

## Abstract

Introduction

The coronavirus disease 2019 (COVID-19) pandemic has affected medical education in many ways. The Association of American Medical Colleges (AAMC) temporarily suspended clinical student rotations, calling for a transition to remote learning. Unfortunately, due to the heavy impact of COVID-19 in our South Florida community, medical students were not able to return to in-person activities for a significant time. During this period, students had remote clerkship learning activities, didactic sessions, narrative projects, and small-group learning sessions, which were front-loaded using Zoom technology (Zoom Video Communications, Inc., San Jose, California, United States) and web-based learning tools. Once in-person clinical experiences resumed, the duration of all third-year clerkships for the remainder of the year was reduced to five weeks to allow for timely graduation. The Herbert Wertheim College of Medicine (HWCOM) Internal Medicine (IM) clerkship has traditionally been an eight-week-long rotation. Other clerkships that varied from six to eight weeks were similarly reduced to five weeks. We hypothesized that the shortened duration of the IM clerkship would have negative impacts on National Board of Medical Examiners (NBME) exam performance and clerkship clinical experiences would likely be affected.

Methods

We compared the NBME subject exam results and end of clerkship evaluations from the Class of 2021 (CO2021) which had the traditional eight weeks of patient care, with the CO2022, which had only five weeks of in-person patient care. A T-test analysis was performed comparing performance on the NBME medicine clinical subject exam between students who completed the usual eight-week rotation versus those who completed a five-week rotation. We also evaluated the IM clerkship course evaluation and analyzed student responses and ratings to assess any areas that were statistically significant when comparing the traditional eight-week IM clerkship to the shortened five-week clerkship.

Results

There was no statistically significant difference (t=0.68, p<0.4951) in mean NBME subject exam performance between cohorts. Students who completed the shortened five-week IM clerkship indicated there was limited volume and diversity of patients, which consequently affected their ability to complete all the required clinical experiences for the IM clerkship. These results indicated a statistically significant difference between the two cohorts (t =3.33, p<.001).

Conclusion

Students with shortened IM clerkship clinical care time (five weeks) were found to have no significant statistical differences in NBME subject exam performance compared to the traditional eight-week cohorts. However, students felt there was a decreased volume and diversity of patients, and they reported greater difficulties in completing the required clinical experiences, with diminished clinical confidence. Time does matter, and clinical time is very valuable for a student's undergraduate medical education. If another pandemic were to arise, the duration of different clerkships should be carefully assessed and individualized, and methods to assess and reclaim lost clinical time during the advanced clinical and postgraduate years should be considered.

## Introduction

The coronavirus disease 2019 (COVID-19) pandemic disrupted medical education starting in March 2020. Due to nationwide limited supplies of personal protective equipment (PPE) and the fear of exposure to a highly contagious virus with limited medical knowledge regarding treatment and prevention, medical schools were advised by the Association of American Medical Colleges (AAMC) to halt clinical rotations [1]. Initial recommendations were to interrupt rotations for only a few weeks, but in our local county, the hospital census for COVID-19 patients remained high for months, and clerkship students were unable to return to clinical duties for five months. This loss of clinical experience for the Florida International University Herbert Wertheim College of Medicine (HWCOM) was very concerning to the clerkship faculty. Although the foundations of medicine can be read and learned, in-person clinical interactions are essential to professional development and are difficult to replace [2]. Learning how to obtain a complete history and perform a correct physical exam are skills that require practice in real time. Paying attention to the daily details of patient care such as monitoring lab results, discussing diagnostic test results at the bedside, and collaborating within a medical team requires active engagement in the interdisciplinary clinical setting. Having adequate time for preceptor-student interactions is important for feedback and role modeling [3].

Because of these clinical closures related to COVID-19, students from the Class of 2022 (CO2022) were significantly restricted in the amount of direct patient contact time. These students only had five weeks of direct patient care compared with the traditional internal Medicine (IM) clerkship at our institution, which is usually eight weeks in length. We were concerned that students would not be able to master the material required to pass the very comprehensive National Board of Medical Examiners (NBME) Medicine subject examination [4]. Prior studies have demonstrated that the pandemic hampered student performance on clinical Objective Structured Clinical Examination (OSCE) examinations [5]. The OSCE is a structured exam using standardized patients where students are observed and assessed taking a history, doing a physical exam, and developing a management plan. Other institutions had similar concerns that shortening the clerkship would affect learning and test performance. To mitigate these concerns, novel teaching methods such as online virtual rounds, modules, and telemedicine were incorporated into the clerkship by different institutions to try to recreate bedside teaching [6-8]. Another concern was whether students would have enough time to complete the required clinical core cases and procedures. 

Several prior studies have concluded that longer Medicine clerkship length is associated with higher clinical subject exam scores, while other studies showed a lack of effect on the NBME score related to clerkship length [9,10]. The aim of the study was to determine whether shortening the IM clerkship time would affect the comprehensive NBME Medicine subject exam performance and also determine if students felt they had enough time to complete the IM clerkship requirements.

## Materials and methods

As in many medical schools across the nation, at Herbert Wertheim College of Medicine, Miami, Florida, students are required to take the standardized NBME Medicine subject exam at the end of their IM clerkship. The Medicine exam is intended to assess knowledge in this area as well as readiness for the United States Medical Licensing Examination (USMLE) Step 2 Clinical Knowledge (CK) exam and is used to assess clinical competency and patient-centered skills required for entrance into residency. Students take the Medicine subject exam on the last day of their clerkship and must attain a minimum required national percentile score to achieve a passing score for the clerkship (in addition to other requirements related to clinical competency). The NBME provides tables with information related to national student performance for the different clerkship lengths.

We set out to compare performance between cohorts on the traditional eight-week IM block versus those on the shortened five-week IM block. These cohorts were selected by the same admission process and were selected by their year of graduation, which affected their curriculum. Students from the Class of 2021 (CO2021) cohort completed the traditional eight-week IM clerkship. Each student completed four weeks of inpatient hospitalist medicine and four weeks of subspecialty elective rotations. The choice of elective rotations included: cardiology, nephrology, infectious diseases, pulmonary/critical care, rheumatology, palliative care, gastroenterology, emergency medicine, and endocrinology. During these eight weeks, students attended didactic sessions once a week that included review topics: cardiology review, NBME question reviews, ethics reflective session, infectious disease review, and chest-X-ray review. Students from the CO2022 cohort completed the shortened five-week IM clerkship that consisted of three weeks of inpatient hospitalist medicine and only two weeks of subspecialty elective. This group had frontloading of didactic sessions during a dedicated pre-clerkship course, which was delivered via Zoom (Zoom Video Communications, Inc., San Jose, California, United States) at the time that clinical sites were unavailable to students due to COVID-19 restrictions. The didactic sessions included some of the same topics traditionally given during the eight-week clerkship, and additional review sessions titled Review of Common IM Cases, Patient Safety, and EKG lecture. CO2021 students on the eight-week schedule were required to complete three Aquifer Internal Medicine cases (Aquifer, Inc., Lebanon, New Hampshire) [11], and encouraged to complete additional cases if so desired, while students in the CO2022 with the shortened five-week clerkship were required to complete 20 Aquifer cases during the clerkship pre-course. These Aquifer online clinical cases help the students learn how to diagnose and treat common medical problems. At the end of the IM clerkship for both cohorts, students were required to complete an internally administered NBME Medicine subject exam, which was composed and graded by the NBME.

Individual student performance on the NBME Medicine subject exam was compared to national student performance and score reports were made available to us by the NBME. Performance on the NBME exam was weighted toward the overall clerkship grade for all students, and performance on the exam was a requirement for delineating clerkship final designation of either Pass, Near Honors, or Honors. The weighting of the NBME exam on final clerkship grade did not vary between the two groups and was set at 30% of their total grade. We used the NBME equated percent correct scores to analyze aggregate student performance for the two cohorts analyzed (CO2021 with the traditional eight-week clerkship length and the CO2022 with the shortened five-week clerkship length).

In addition to comparing performance on the NBME Medicine subject exam, we also analyzed student responses on the end-of-clerkship satisfaction surveys. As a professional competency, students are required to complete this clerkship satisfaction evaluation at the end of every rotation. The clerkship evaluation questionnaire completed by students contains 12 variables related to the student’s experiences during the clerkship. Clerkship evaluation items use a five-point Likert-type rating scale where a rating of 5 equals Strongly Agree, and a rating of 1 equals Strongly Disagree. The survey questions were the same for both cohorts and included items pertaining to multiple aspects of their experiences with the rotation at their assigned sites (volume of patients, diversity of patients, active participation in the management of patient care, opportunity to develop differential diagnoses, clerkship team evaluations, and individual preceptor evaluations, etc.). Many of the variables in the evaluation relate to the ability to complete procedures, satisfaction with the assigned schedule, and orientation. Specifically, did the volume and diversity of patients seen allow the student to achieve the clerkship's learning objectives? Students are required to see certain core cases during their rotation and these core cases include common IM diagnoses such as diabetes and hypertension. We compared the IM clerkship course evaluation ratings and analyzed student responses to identify any areas that were statistically significant when comparing the traditional eight-week clerkship to the shortened five-week clerkship. The goal was to see if any differences existed between the two cohorts.

An independent t-test analysis was conducted to compare: (1) NBME subject exam aggregate results for each cohort and (2) end-of-clerkship evaluations for each cohort. The effect size was also analyzed using Cohen’s D as a measure for each analysis.

## Results

T-test analysis was performed comparing performance on the NBME exam between students who completed the usual eight-week clerkship (n=117) versus those who completed the shorter five-week clerkship (n=118). There was no statistically significant difference (t=0.68, p = 0.4591) in mean NBME subject exam scores between the two groups. The effect size shown in Table 1 indicates a small effect (d=.08) for Cohen's analysis (1988) [12].

**Table 1:** T-test: Comparison of NBME IM performance between eight-week clerkship (CO2021) and five-week clerkship (CO2022) Diff (1-2) = 0.7541; Pooled STD = 8.45810; Effect Size (Cohen’s D) = 0.0892 NBME: National Board of Medical Examiners; CO2021: class of 2021; CO2022: class of 2022; IM: internal medicine

Group	N	Mean	STD	Low Score	High Score	P-value	Result
Five-week clerkship (CO2022)	118	75.7712	8.7953	52	100	0.4591	No significant difference
Eight-week clerkship (CO2021)	117	75.0171	8.1038	58	100		

We reviewed anonymous summative end-of-clerkship student evaluation comments to assess student satisfaction during the five-week and eight-week rotations. Based on student responses, two areas appear to have been particularly impacted by shortened clerkship length. One was the opportunity to evaluate undiagnosed patients and develop a differential diagnosis, and the other was the volume and diversity of patients seen. 

The students who completed the five-week IM clerkship indicated there was a limited volume and diversity of patients, which consequently affected their ability to meet all the required clinical experiences for the IM clerkship. The results reveal a statistically significant difference between the two cohorts (t=3.33, p <.001). The effect size for this analysis indicates a medium effect (d=0.435) for Cohen's analysis (Table 2). 

**Table 2:** T-test: Comparison of IM end-of-clerkship evaluation (Question 4: Volume and diversity of patients allowed me to achieve the clerkship's learning objectives) between eight-week clerkship (CO2021) and five-week clerkship (CO2022) Diff (1-2) = 0.3162, Pooled STD = 0.72629, Effect Size (Cohen’s D) = 0.4354 CO2021: class of 2021; CO2022: class of 2022; IM: internal medicine

Group	N	Mean	STD	Minimum	Maximum	P-value	Result
Five-week clerkship (CO2022)	117	4.6923	0.5642	1	5	0.001	Significant difference.
Eight-week clerkship (CO2021)	117	4.3761	0.8583	1	5		

## Discussion

The practice of medicine entails more than a basic fund of knowledge. Answering questions successfully on a written exam is important, but may not necessarily translate into confident patient care. The ability to communicate effectively with patients, families, and other medical professionals, to work as a member of a medical team, and to acquire organizational skills may not be readily reflected by a standardized examination score. Patient interactions are essential to the development of a physician/healthcare provider and should continue to play an important role in the evaluation of competency. Due to COVID-19 restrictions, all clerkships at our institution were allotted a five-week duration to allow for timely student graduation. The impact this may have had on longer clerkships such as the IM and Surgery could be significant. 

The results of the current study suggest that five weeks of clinical time with front-loading of valuable information provides an adequate foundation of knowledge for students rotating in IM. The NBME medicine subject exam is a very comprehensive examination that has been shown to provide a good assessment of medical knowledge. Both cohorts had comparable scores and performance in the subspecialty areas. This lack of effect on NBME subject exam performance has also been previously described for other subject examinations [12]. The study by Alexandraki et al. also suggests that shortened IM clerkship time due to COVID-19 closures did not objectively affect exam performance on the NBME Medicine subject exam [13]. However, it did have other effects on medical students as they struggled to see the required clinical experiences and diversity of cases during the shortened time period. Some students reported that their overall experiences were insufficient, and this was concerning, as the longitudinal clinical clerkships are fundamental to the development of clinical and professional skills for future resident physicians. These students were exposed to a lower volume and diversity of patients, as a large number of patient hospitalizations were related to COVID-19, and elective tests and hospitalizations were restricted. Hospital beds were also restricted and patients were discouraged to visit the emergency room for evaluation unless they had life-threatening emergencies. During the height of the COVID-19 pandemic, the majority of medical schools did not allow students to directly care for COVID-19 patients. Many patients that were admitted for common medical problems such as cardiac decompensation and unstable angina were incidentally found to test positive for COVID-19, and this further limited clinical interactions for students. As a result, in addition to the shortened clerkship time, students experienced a reduction in the diversity and volume of patients they would normally be exposed to, as well as reduced bedside teaching hours [14]. This is of paramount importance, as prior research has shown that student performance improves as students see a greater number of patients per day [15].

IM is considered the foundation for many specialties and should be given enough dedicated clinical time to expose students to a variety of diverse patients [16]. Student psychosocial well-being is also paramount, and we should consider minimizing unnecessary stress as a priority in healthcare education [17]. While it is generally accepted that a healthy young student should not develop severe disease if infected with a virus such as severe acute respiratory syndrome coronavirus 2 (SARS-CoV-2) [18], it is also important to protect these essential members of the healthcare team that will be our future healthcare leaders. As clinical educators, we were trying to protect students from COVID-19 exposure, but many students expressed their desire to learn and treat this disease. They would soon be interns on medical teams with significant patient responsibilities and wanted to know how to treat patients with COVID-19. Studies have shown that virtual rounding and telemedicine have been well received by students and provide valuable exposure, but cannot be a substitute for direct patient care [[19-21].

As medical educators, we will need to continue to evaluate the dilemma of providing essential clinical patient exposures while protecting student physical and emotional well-being. Our retrospective study demonstrated that while students can learn complex medical information and successfully pass a comprehensive standardized NBME examination in a shortened time period when provided with information remotely, exposure to diverse patient volumes and experiences was restricted, and students did not feel fully confident after their shortened experience. Follow-up evaluations are necessary to assess if this missed clinical time can be reclaimed during the final clinical year and postgraduate training. Assessment of psychological well-being in the form of self-reported graduate student and resident questionnaires is important and we encourage these future investigations. As COVID-19 cases continue but illness appears less severe, students across the country are taking a more active role in the care of patients with this infection. Investigation of psychological well-being while caring for these patients is also important to assess. 

We recognize there are limitations to our study. The cohorts were from one institution and geographical area that had specific restrictions on students returning to the clinical setting. We also realize the cohorts were non-randomized. We are confident that future research and technological efforts will assist with innovations in medical education for any future pandemic that may arise.

## Conclusions

The COVID-19 pandemic has affected medical education in many ways. When comparing student performance and satisfaction during the traditional eight-week IM clerkship to that during the shortened five-week IM clerkship, both cohorts had similar performances on the national NBME Medicine subject exam. However, students with reduced clerkship time did not feel as clinically confident. Significant and diverse clinical exposures are essential to provide the skills needed to become a competent physician. Time does matter, and clinical time is very valuable for a student's undergraduate medical education and development as a provider of care.
